# Complex dynamics of glutamate-induced calcium responses in astrocytes from the nucleus of the solitary tract of mice

**DOI:** 10.1590/1414-431X2025e15017

**Published:** 2026-01-30

**Authors:** M.M. de Souza, L.R. Zacharias, R.M. Leão

**Affiliations:** 1 Departamento de Fisiologia, Faculdade de Medicina de Ribeirão Preto, Universidade de São Paulo, Ribeirão Preto, SP, Brasil; 2 Departamento de Neurociências e Ciências do Comportamento, Faculdade de Medicina de Ribeirão Preto, Universidade de São Paulo, Ribeirão Preto, SP, Brasil

**Keywords:** Astrocytes, Calcium, Glutamate, Nucleus of the solitary tract

## Abstract

Astrocytes play critical roles in the physiological responses of the central nervous system (CNS). Located near pre- and postsynaptic sites, they detect released neurotransmitters and gliotransmitters that modulate neuronal function. Astrocytic responses to neurotransmitter excitation often involve increases in cytoplasmic calcium, triggering the release of gliotransmitters that further influence neuronal activity. The nucleus of the solitary tract (NTS), a brainstem region integrating diverse physiological functions such as cardiovascular, respiratory, digestive, and metabolic reflexes, is modulated by astrocytic activity. To better understand the dynamics and diversity of calcium responses in NTS astrocytes to the primary excitatory neurotransmitter glutamate, we investigated individual subpostremal NTS astrocytes in brainstem slices from mice using the calcium fluorescent dye Fluo-4. We observed that only a subset of astrocytes exhibited an increase in cytoplasmic calcium in response to glutamate, while a smaller fraction showed a decrease in cytoplasmic calcium. Interestingly, in the presence of tetrodotoxin, which inhibits action potentials, the proportion of astrocytes with increased calcium levels was halved, and most astrocytes instead exhibited decreased calcium levels. Further analysis revealed that response peaks were correlated with total calcium levels after glutamate application, whereas response latencies and widths of positive calcium signals were not correlated with peak values. Negative peaks have distinct kinetics from positive peaks, corroborating that they represent different processes. These findings demonstrate that NTS astrocytes constitute a heterogeneous population with diverse responses to extracellular glutamate, highlighting their complexity in modulating brainstem functions.

## Introduction

It is already well established in the literature that astrocytes play an active role in the physiology of the central nervous system (CNS), not a mere supporting role. Astrocytes have a high adaptive plasticity and are heterogeneous in form and function, indispensable for proper functioning of the CNS (1). Unlike neurons, astrocytes do not generate action potentials; instead, they use changes in intracellular calcium (Ca^2+^) as a cellular signal of their activation. Thus, the cellular excitability of astrocytes is based on the variation of Ca^2+^ concentration in the cytosol. Such Ca^2+^ oscillations either arise spontaneously or are induced by neurotransmitters (2,3). Astrocytes, when stimulated by glutamate, generate an increase of Ca^2+^ in the cytosol, which is primarily dependent on calcium release from intracellular stores (1,4) and generates responses leading to the release of gliotransmitters and modulation of neuronal activity (5,6), which is the concept of the tripartite synapse (2).

One of the regions where the neurophysiological roles of astrocytes have been broadly studied in recent times is the nucleus of the solitary tract (NTS). The NTS, located in the brainstem, is a site of visceral information integration, receiving afferent inputs from cranial nerves (vagus, facial, and glossopharyngeal nerves) that form the solitary tract (ST). The information that arrives at the NTS is related to the control of several reflexes, such as respiratory, cardiac, vascular function, gastrointestinal, baroreflex, and chemoreflex (7-10). The subpostremal NTS is an area that receives terminals from several organs and mediates different autonomically mediated reflexes (10-12). Several studies show the importance of NTS astrocytes in the regulation of energy balance and other autonomic functions (13-18). Electron microscopy studies indicate that 50-60% of NTS terminals are covered by astrocytic processes, suggesting a substantial overlap between astrocytes and terminals (19).

Glutamate is the CNS's primary excitatory neurotransmitter and is released by the terminals of the solitary tract. NTS astrocytes express AMPA, NMDA, and mGlu receptors (1,20-22), and it has been reported that glutamate activates NTS astrocytes through ionotropic AMPA receptors (22), generating an increase in cytoplasmic calcium by calcium influx through calcium-permeable AMPA receptors.

Although they are usually treated as a homogeneous population, astrocytes are known to be a heterogeneous cellular population, even within the same brain regions (1,23,24). They can express distinct gene profiles, ion channel compositions, gap junction connectivity, and membrane potential, creating a functional specialization of astrocytes. Based on the relevance of astrocytic signaling in modulating the autonomic reflexes of the NTS, we decided to investigate the profile of NTS astrocytes in response to glutamate stimulation in more detail to identify a possible heterogeneity of NTS astrocytes in response to applied glutamate. Thus, the objective of this work was to analyze the activation profile of subpostremal NTS astrocytes by glutamate using Ca^2+^ imaging and to analyze the whole population of astrocytes of this region to detect functional differences in NTS astrocytes related to the response to glutamate.

## Material and Methods

### Animals

The Ethics Committee on the Use of Animals (CEUA) from the School of Medicine of Ribeirão Preto (Brazil) approved the experimental procedures under protocol 135/2019. We used C57Bl/6 male mice aged between 4 and 8 weeks, which were housed in appropriate cages and kept in an acclimatized room (21-22°C) in the animal house of the School of Medicine of Ribeirão Preto, University of São Paulo (FMRP-USP) under a 12-h light/dark cycle with food and water *ad libitum*. All experiments were performed at the Laboratory of Neurophysiology and Synapse of the Department of Physiology of the FMRP-USP.

### Solutions

Artificial cerebrospinal fluid (aCSF) for cutting and preparing brainstem slices (mM) was: 87 NaCl, 2.5 KCl, 1.25 NaH_2_PO_4_, 25 NaHCO_3_, 75 sucrose, 25 D-glucose, 0.2 CaCl_2_, 7 MgCl_2_, with 330 mOsmol/kg H_2_O and pH 7.4 when bubbled with the carbogenic mixture (95%O_2_/5%CO_2_).

Recording aCSF solution (mM): 125 NaCl, 2.5 KCl, 1.25 NaH_2_PO_4_, 25 NaHCO_3_, 5 sucrose, 5 D-glucose, 2 CaCl_2_, 1 MgCl_2_, with 290 mOsmol/kg H_2_O and pH 7.4 when bubbled with the carbogenic mixture (95%O_2_/5%CO_2_).

### Preparation of slices

Mice were deeply anesthetized with isoflurane and decapitated. The brain was quickly removed and kept in a Petri dish containing ice-cold (4°C) cutting aCSF solution, previously bubbled with the carbogenic mixture, to separate the brainstem. These tissue slices containing the subpostremal NTS and surroundings (±200 µm rostral and caudal) were obtained using a Vibrating Microtome (Compresstome VF-300-0Z, USA). The slices were incubated at 32-33°C for 45 min in recording aCSF solution and subsequently at room temperature.

### Labeling and identification of astrocytes

Selective labeling with sulforhodamine 101 (SR101; 1 µM Sigma-Aldrich^®^, USA) was used to identify the astrocytes in the NTS. SR101 was added to the aCSF and incubated for 45 min as previously described (25-27). Labeled astrocytes were identified by red fluorescence when excited by a wavelength of 586 nm using an LED illuminator (Excelitas, Canada).

### Calcium imaging

Changes in the intracellular concentration of Ca^2+^ indicate astrocyte activity. Intracellular Ca^2+^ was monitored by epifluorescence microscopy using the calcium indicator Fluo-4-AM (Thermo Fisher Scientific^®^, USA). After 45 min at 32-33°C, the slices were incubated in a Petri dish containing 2 mL of the recording solution and 2-5 μL of Fluo-4-AM (final concentration: 2-10 μM) with 0.01% pluronic acid for approximately 30 min, bubbled with the carbogenic mixture. The uptake of Fluo-4-AM is selectively preferred by astrocytes in this time window (26), which we confirmed by co-labeling with SR-101.

Then, the slices were transferred to a chamber positioned under an upright optical microscope (Olympus, Japan) and perfused with aCSF at the rate of 1 mL/min (regular pathway). Astrocytes were visualized with a 60× immersion objective (BX51WI, Olympus). Ca^2+^ signals were recorded using a PCO edge CMOS camera (PCO) attached to the microscope. The slices were illuminated for 200 ms with an LED (X-Cite, Excelitas) at 494 nm, using an emission filter for FITC, and the images were acquired every 1 s.

Drugs were applied through the regular perfusion pathway, and L-glutamate was applied using a borosilicate capillary positioned close to the objective to ensure fast and local delivery of the transmitter (“fast” pathway) at the rate of 2 mL/min).

### Drugs and reagents

Isoflurane (Isoforine^®^) was purchased from Cristália Produtos Químicos Farmacêuticos (Brazil). L-Glutamic acid monosodium salt monohydrate (Sigma-Aldrich) was diluted directly in the recording solution. Stock solution of tetrodotoxin (TTX; Alomone Labs, Israel) was prepared in ultrapure H_2_O and diluted in the aCSF at the final concentration of 0.5 µM. Sulforhodamine 101 (SR101; Sigma-Aldrich) was incubated with the slices at a concentration of 1 µM. Fluo-4-AM (Thermo Fisher Scientific) was diluted in DMSO with 20% Pluronic acid (Sigma-Aldrich).

### Data analysis

The 2 to 3 slices containing the subpostremal NTS were recorded and analyzed. The frames were analyzed using ImageJ software (NIH, USA) to merge the 600 recorded frames and create a video with astrocytic calcium oscillations. Astrocytes were identified, and regions of interest (ROIs) corresponding to the cell bodies were delimited. A custom MATLAB (The MathWorks, USA) script was used to extract the average fluorescence intensity of each ROI as a function of time, accounting for photobleaching and background subtraction, and estimate the individual relative fluorescence (ΔF/F0), where F0 corresponds to a 30-s baseline fluorescence and ΔF as the difference of raw fluorescence and baseline fluorescence (F-F0). Individual relative fluorescence (F/F0) was convoluted with a Gaussian function using a 5-s window, and then z-scored against the baseline, resulting in a z-scored F/F0 (28). Astrocytes were classified as not modulated, negatively modulated, or positively modulated by averaging the peak z-scored F/F0, where astrocytes with average z-scored F/F0 peaks higher than two or lower than -2 during experimental intervals were considered positively or negatively modulated, respectively, and astrocytes with average peaks z-scored F/F0 between to -2 and 2 were considered not modulated. Then, the fluorescence latency to the peak, full-width at half maximum (FWHM), and the area under the curve (AUC) were calculated.

### Experimental design and statistical analyses

Statistical analyses were performed with custom MATLAB scripts and GraphPad Prism version 8.0 (GraphPad^®^ Software, USA). The Kolmogorov-Smirnov test was used to assess the normality of variable distribution. We used the Mann-Whitney non-parametric test for comparing two variables and the Kruskal-Wallis non-parametric test with the uncorrected Dunn's test for comparing three or more variables. P-values <0.05 were considered statistically significant.

## Results

### Identifying astrocytes and the effect of change in perfusion velocity on calcium fluorescence

**Figure 1:**
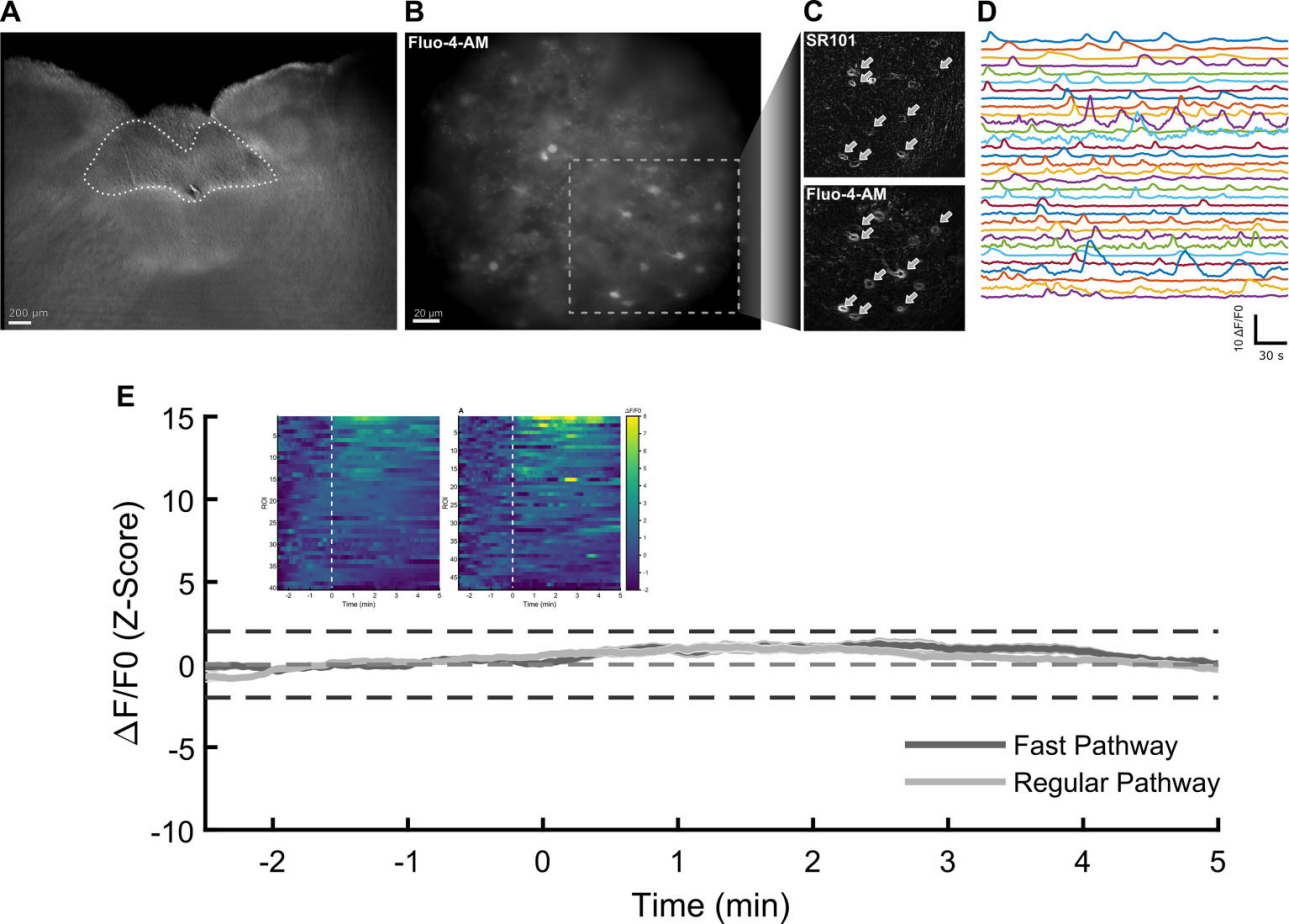
Identification of nucleus of the solitary tract (NTS) astrocytes and modulation of astrocyte activity by perfusion via different pathways. **A**, Representative slice of C57BL/6 mouse with the subpostremal NTS (delineated) visualized with Differential Interference Contrast (DIC) microscopy (scale bar: 200 µm). **B**, Subpostremal NTS region showing Fluo-4-AM labeled astrocytes (scale bar: 20 µm). **C**, Zoom of panel B comparing astrocyte labeling with SR101 (top) and Fluo-4-AM (bottom); white arrows represent the same cell co-stained with both dyes. **D**, Examples of calcium oscillations recorded in Fluo-4-AM labeled astrocytes. **E**, Mean relative fluorescence (ΔF/F_0_) (z-scored) of astrocytes with regular pathway (light grey) and with fast pathway (dark grey) during baseline (-3 to 0 min) and after pathway transition (0 to 5 min ). Dotted horizontal lines represent the ±2 SD of the Z-scores. Inset plots show individual astrocyte ΔF/F_0_ before and after (dashed-white line) pathway transition with regular pathway (left) and fast pathway (right).

Astrocytes were identified by double labeling with SR101 and Fluo-4 (Figure 1A-C). Our Fluo-4 labeling protocol effectively labeled astrocytes preferentially; thus, we did not perform SR101 labeling in all experiments to avoid possible effects of SR101 on astrocyte activity. The Fluo-4-labeled cells presented calcium oscillations typical of astrocytes (Figure 1D), and calcium fluorescence was stable during the recording period (40 cells, 5 slices from 3 animals, Figure 1E left inset plot and “regular pathway”). To test whether the change in perfusion rate, achieved by using the fast and localized pathway we employed for applying glutamate, altered calcium fluorescence by activating mechanoreceptors present in astrocytes (23), we measured fluorescence after perfusing aCSF through the fast pathway. Switching from the regular pathway to the fast and localized perfusion system applying only aCSF did not modulate the average fluorescence activity above (or below) our threshold of two standard deviations of the z-scored ΔF/F0, with similar dynamics compared to the regular pathway (Figure 1E). Activity of individual astrocytes showed significant modulation of astrocytes after switching to the fast pathway, with only 10% of cells being positively modulated during application of aCSF by the fast pathway (n=5 of 49, 6 slices from 3 animals, Figure 1D right inset plot), with only one single cell showing a significant negative modulation, thus indicating that changes in perfusion rate systems do not significantly affect calcium fluorescence.

### Glutamate application generated diverse responses in astrocytes

**Figure 2:**
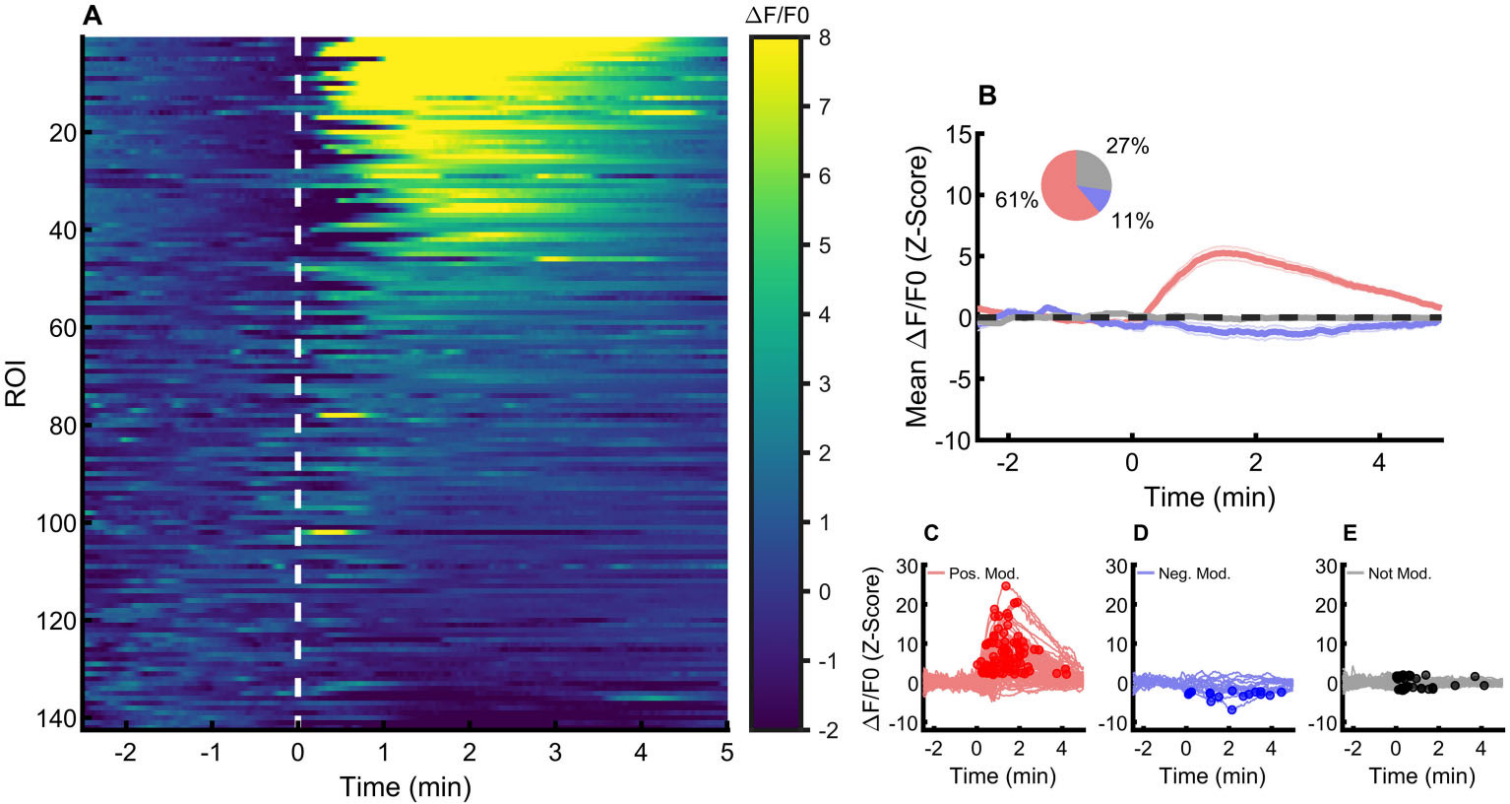
Cytoplasmatic calcium changes in response to glutamate. **A**, Relative fluorescence (ΔF/F_0_) (z-scored) of individual astrocytes before (-3 to 0 min) and after (0 to 5 min) glutamate perfusion. The dashed white line represents the onset of glutamate perfusion. **B**, Mean and SEM ΔF/F_0_ of positively (red), negatively (blue), and not modulated astrocytes (light grey) before and after glutamate perfusion. The inset shows the proportions of each response. **C**, Individual ΔF/F_0_ (z-scored) of positively modulated astrocytes before and after glutamate perfusion. **D**, Individual ΔF/F_0_ (z-scored) of negatively modulated astrocytes. **E**, Individual ΔF/F_0_ (z-scored) of not modulated astrocytes. Circles represent the identified peak of ΔF/F_0_ activity after glutamate perfusion. ROI: regions of interest.

L-glutamate (500 µM) was applied to the slices through the fast pathway. We recorded the alterations of cytoplasmic calcium in 142 NTS astrocytes from 14 slices (7 animals). All responses are shown in Figure 2A. Most of the astrocytes responded with an increase in the fluorescence above two standard deviations of the z-scored ΔF/F0 (88 of 142, 66%; Figure 2B and C). Forty astrocytes (27%) did not present calcium oscillations above or below two standard deviations of the z-scored ΔF/F0 and were classified as non-responsive (not modulated, Figure 2B and E). Surprisingly, 11% of the astrocytes (16) presented negative calcium oscillations below our threshold of -2 standard deviations of the z-scored ΔF/F0 (Figure 2B and D).

### Inhibition of action potential firing changed the astrocytic response to glutamate

Because applied glutamate can trigger action potential firing in neurons that can induce release of synaptic glutamate and other transmitters, which can affect the observed response, we applied glutamate in the presence of the action potential blocker tetrodotoxin (TTX) to measure the response of astrocytes, with no interference from synaptic released transmitters (glutamate, GABA, or ATP).

**Figure 3:**
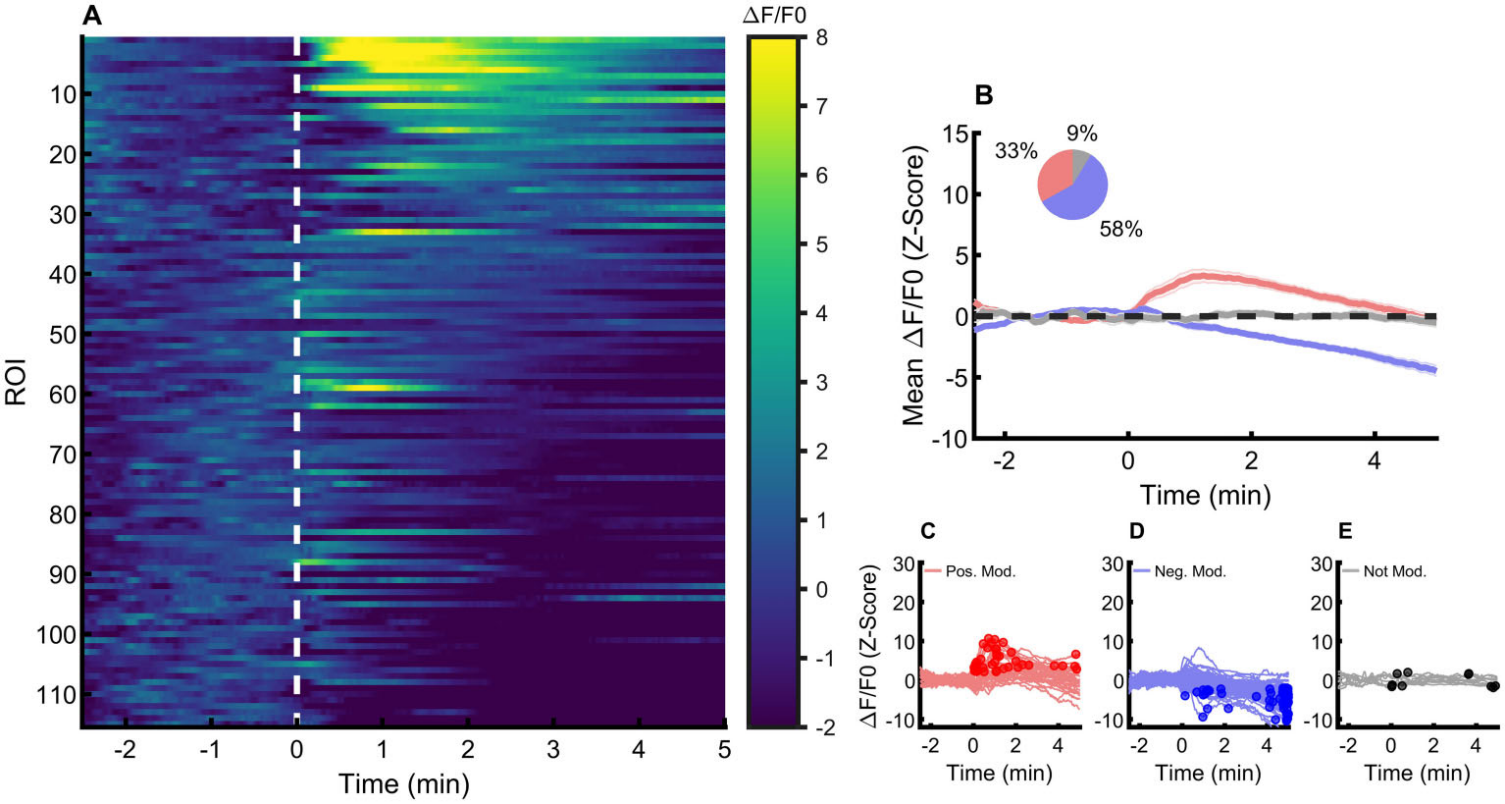
Cytoplasmatic calcium changes in response to glutamate in the presence of tetrodotoxin (TTX). **A**, Relative fluorescence (ΔF/F_0_) (z-scored) of individual astrocytes before (-3 to 0 min) and after (0 to 5 min) glutamate perfusion. The dashed white line represents the onset of glutamate perfusion. **B**, Mean and SEM ΔF/F_0_ of positively (red), negatively (blue), and not modulated astrocytes (light grey) before and after glutamate perfusion. The inset shows the proportions of each response. **C**, Individual ΔF/F_0_ (z-scored) of positively modulated astrocytes before and after glutamate perfusion. **D**, Individual ΔF/F_0_ (z-scored) of negatively modulated astrocytes. **E**, Individual ΔF/F_0_ (z-scored) of not modulated astrocytes. Circles represent the identified peak of ΔF/F_0_ activity after glutamate perfusion.

We recorded 115 astrocytes from 14 slices (8 animals) by applying glutamate with TTX and found a decrease in half of the positive-modulated astrocytes (n=38, 33%). Surprisingly, the proportion of negative-modulated astrocytes increased to 58% (n=67), and the proportion of not-modulated astrocytes decreased to 9% (n=10) (Figure 3).

We concluded that, contrary to our expectations, most astrocytes in NTS did not respond with a cytoplasmic calcium increase after application of glutamate, and a sizable fraction of cells did respond to glutamate application with a decrease in calcium.

### Positive and negative peaks correlated with the global changes in calcium after glutamate

**Figure 4:**
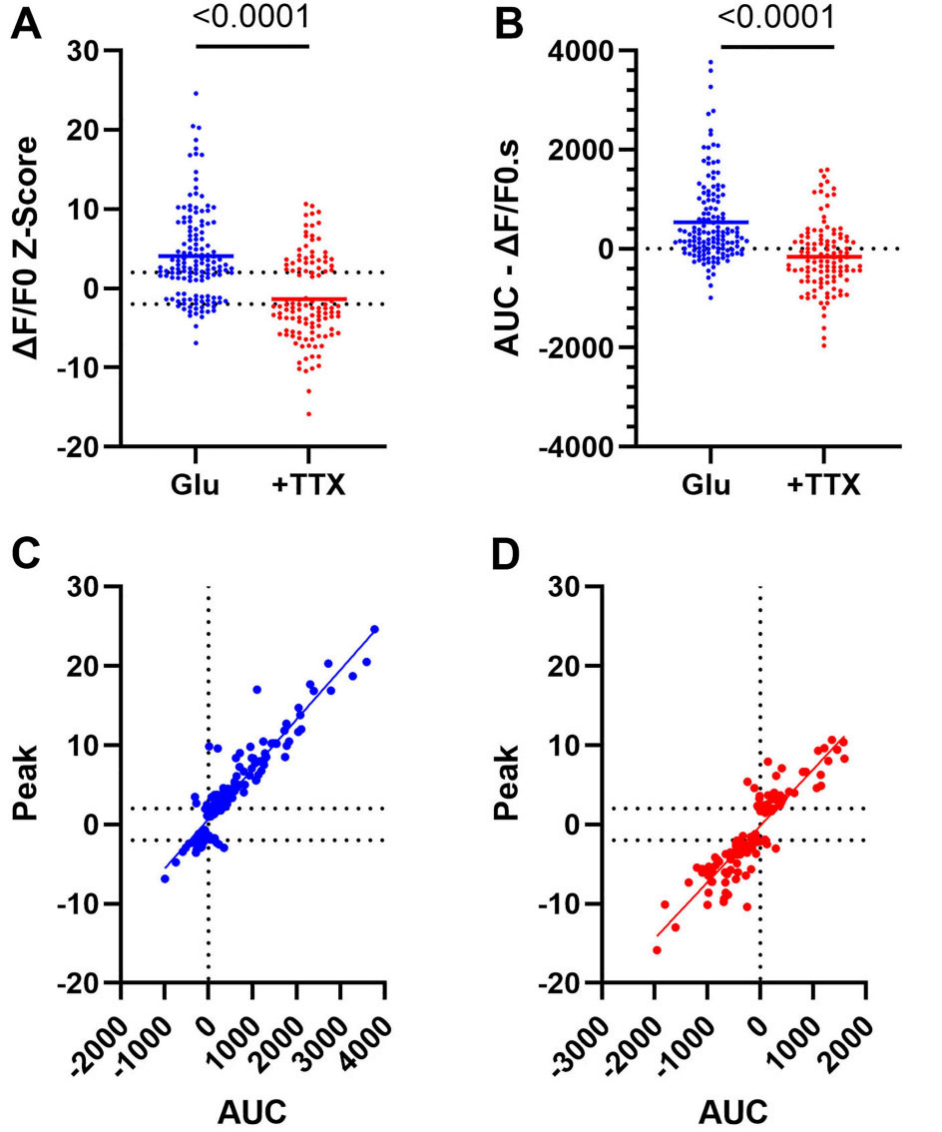
Peak and area under the curve (AUC) of calcium events. **A**, Calcium peaks (relative fluorescence (ΔF/F_0_) z-scored) of astrocytes in response to glutamate (GLU) and glutamate plus tetrodotoxin (+TTX). **B**, AUC (ΔF/F_0._s) of the calcium responses of astrocytes in response to glutamate and glutamate plus TTX. **C**, Correlation of peaks and AUCs (glutamate); **D**, Correlation of peaks *vs* AUCs (glutamate + TTX). Dotted horizontal lines represent the ±2 SD of the Z-scores. Simple linear regression was used for analysis of correlation.

We then analyzed the peak responses of both groups of astrocytes. The distribution of all peaks (both negative and positive) exhibited a broad amplitude range (Figure 4A and B). The mean peak amplitude (ΔF/F0-z-score) was 4±0.5 for glutamate only and -1.4±0.5 for glutamate + TTX (P<0.0001). This difference reflects the different proportions of positive and negative modulated cells in both groups. Because an occasional transient change in calcium could cause the observed peak fluorescence, we measured the area under the curve (AUC) of fluorescence, which provides a more consistent measurement of the variation in cytoplasmic calcium during the 5-min recordings. Again, we found a similar distribution of AUCs, with a mean positive AUC after glutamate and a mean negative AUC in response to glutamate + TTX (Figure 4C and D). A correlation analysis between peaks and AUCs revealed a strong correlation between the two parameters in both groups (glutamate: R^2^=0.86, P<0.0001; glutamate + TTX: R^2^=0.8, P<0.0001). We concluded that calcium peaks, both positive and negative, are correlated to the total change in calcium caused by glutamate and are not random fluctuations of cytoplasmic calcium.

### Features of negative and positive responses

**Figure 5:**
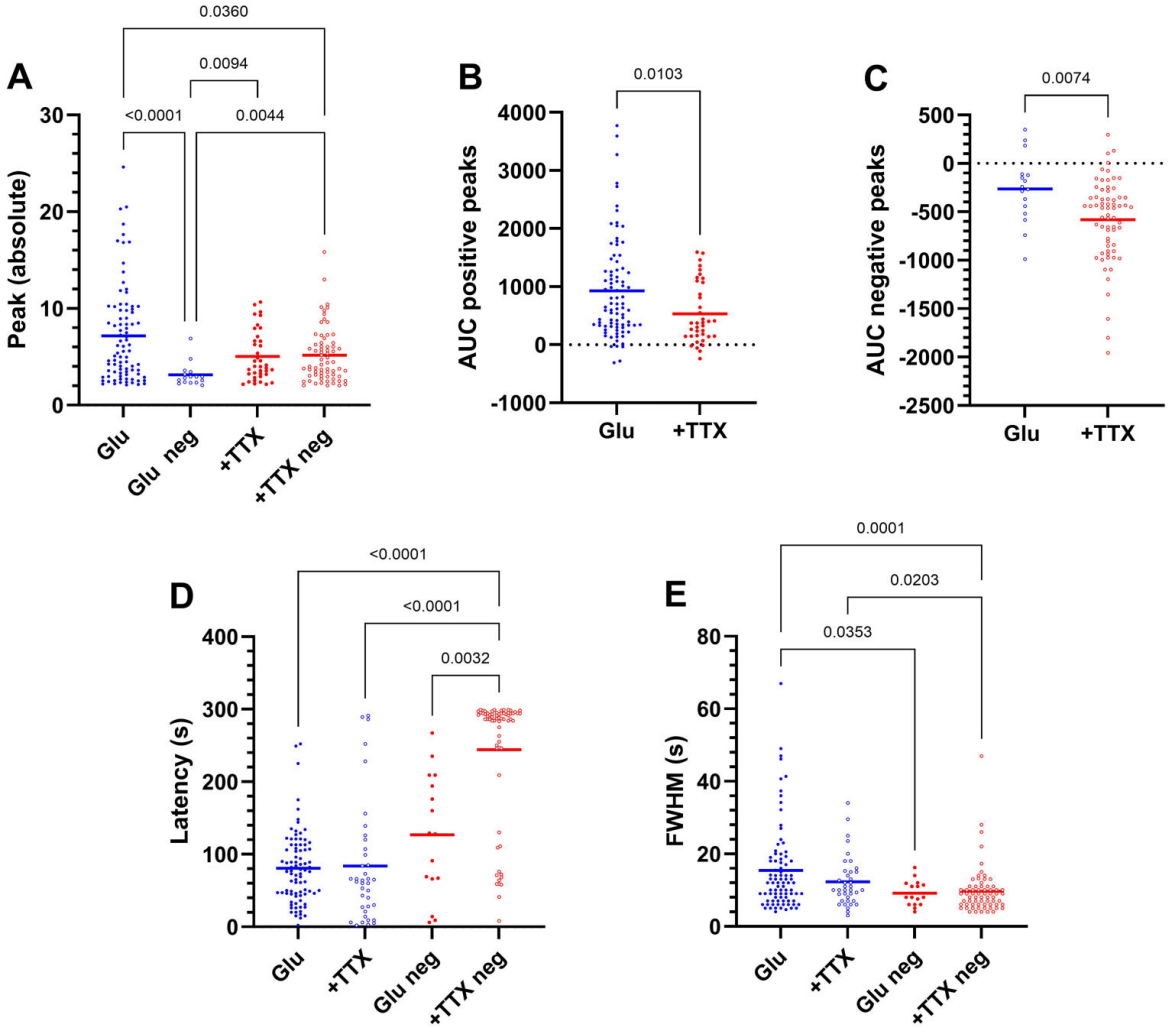
Peak, latencies, and full-width at half maximum (FWHM) of positive and negative events. **A**, Absolute peaks. **B**, Area under the curve (AUC)s (positive peaks). **C**, AUCs (negative peaks). **D**, Latencies (seconds). **E**, Full-width at half maximum (seconds). GLU: glutamate; TTX: tetrodotoxin.

We analyzed positive and negative peaks separately. The amplitude of positive peaks was similar between glutamate and glutamate + TTX conditions (P=0.054), but significantly larger than the absolute amplitude of negative peaks in both conditions (P<0.0001). Moreover, the absolute negative peaks induced by glutamate were significantly smaller than the positive peaks (P<0.0001). Interestingly, positive peaks in the presence of TTX were comparable in absolute amplitude to negative peaks in TTX (Figure 5A).

The AUC of positive calcium events was greater in response to glutamate alone. In contrast, the AUC of calcium events related to negative peaks was greater in the presence of TTX. Notably, a few cells exhibited positive peaks with negative AUCs, and vice versa (Figure 5B and C).

Negative peaks in TTX showed significantly longer latencies (Figure 5D). Additionally, the FWHM of negative calcium events was significantly smaller (Figure 5E).

**Figure 6:**
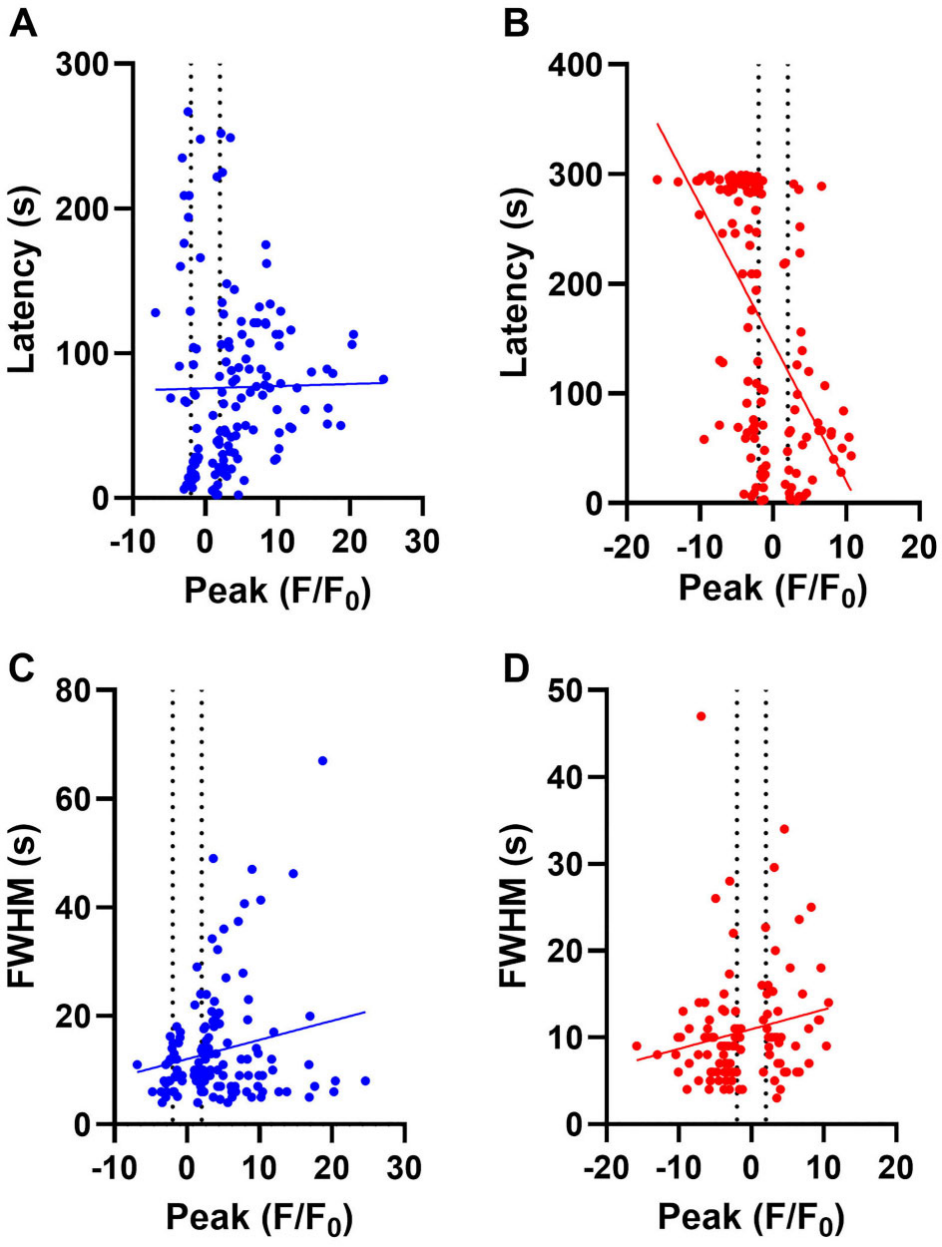
Correlation of latencies and full-width at half maximum (FWHM) with peaks. **A**, Latency *vs* peaks (glutamate); **B**, latency *vs* peaks (glutamate + tetrodotoxin (TTX)); **C**, FWHM *vs* peaks (glutamate); **D**, FWHM *vs* peaks (glutamate + TTX). Vertical dotted lines represent the ±2 SD of the Z-scores. Simple linear regression was used for analysis of correlation.

Latencies and peaks (Figure 6A and B) showed significant negative correlations only in TTX (Figure 6B; P<0.0001, r^2^=0.3). FWHM correlated very weakly with peaks in glutamate (P=0.02; r^2^=0.02) and in glutamate + TTX (P=0.049; r^2^=0.03) (Figure 6C and D).

These findings suggest that positive and negative peaks represent distinct phenomena, as evidenced by their differences in amplitude and kinetics.

## Discussion

Astrocytes have been shown to participate in several physiological reflexes mediated by the NTS (13,15,29-33). These roles are known to be mediated by the glutamate release of solitary tract terminals. In this work, we aimed to understand the diversity of responses of NTS astrocytes to glutamate, their primary excitatory neurotransmitter. For this, we perfused Fluo-4-labeled astrocytes with glutamate to measure the changes in cytoplasmic calcium caused by excitation with this neurotransmitter in all identified astrocytes in the slice.

We used a labeling protocol that was previously demonstrated to be specific for astrocytes (25-27) and confirmed that this Fluo-4 labeling protocol in NTS was specific for astrocytes by co-labeling with the astrocytic marker SR101 (34). Fluo-4-labeled cells presented calcium oscillations typical of astrocytes. Additionally, electrophysiological recordings performed in NTS astrocytes labelled using the same labelling protocol demonstrated that the labelled cells had the electrophysiological profile of astrocytes (M.S. da Luz, D. Accorsi-Mendonça, and B.H. Machado, unpublished results, with permission; Supplementary Figure S1). However, in some slices, we observed occasional large, apparently unlabeled cells expressing weak fluorescence after glutamate treatment. We believe these cells are probably neurons, and we did not use them for analysis. Nevertheless, because SR101 is not specific for astrocytes, it is possible that some labeled cells could be oligodendrocytes (35).

We chose to use a concentration of 500 µM of glutamate, similar to that observed in the extrasynaptic space by cerebral astrocytes after synaptic stimulation (0.29 mM) (36). Additionally, we employed a method that normalized each astrocyte's fluorescence by its baseline Z-score, thereby minimizing individual fluorescence fluctuations and changes in baseline fluorescence during recording, such as photobleaching, to obtain more stable baseline values and detect changes more efficiently.

Using this approach, we demonstrated that NTS astrocytes exhibit heterogeneous responses to glutamate application. The observed reactions ranged from strong calcium elevations to weak calcium signals, with a notable subset of astrocytes showing a decrease in cytoplasmic calcium in response to glutamate.

Inhibiting action potential firing with TTX significantly reduced the size and amplitude of the calcium response, as well as the number of astrocytes that were positively modulated. This finding suggests that a portion of the astrocytic response measured in Figure 2 could be mediated by neuron-released glutamate rather than by the applied glutamate, or by the involvement of other synaptic transmitters, such as GABA and ATP, as astrocytes express both GABAergic and purinergic receptors (21). It is possible that astrocytic AMPA receptors may be located near release sites and are more effectively activated by synaptic-released glutamate than by perfused glutamate. We used a high concentration of glutamate, exceeding levels typically present outside the synaptic cleft after synaptic stimulation (36). Thus, we believe that we effectively activated astrocytic glutamate receptors. Further experiments using rapid glutamate release methods, such as pressure application or glutamate uncaging, and testing other neurotransmitters released by NTS synapses, like GABA and ATP, are needed to clarify the mechanisms of this effect of TTX.

A significant proportion of non-responsive astrocytes suggests that most NTS astrocytes do not express glutamatergic receptors and possibly do not sense glutamate release. On the other hand, unresponsive cells can generate calcium signals in the processes and not in the cell body in response to glutamate application, since these events are not necessarily correlated (37).

Our most unexpected finding was that several NTS astrocytes did not exhibit an increase in cytoplasmic calcium in response to glutamate stimulation. Instead, a significant subset responded with a decrease in intracellular calcium. These astrocytes were viable, as they took up Fluo-4 and displayed calcium oscillations before glutamate application (Figures 2 and 3). Although increases in astrocytic cytoplasmic calcium are usually associated with activation of astrocytes by neurotransmitters, it has been shown that several astrocytes in the ventral tegmental area respond to dopamine D2 receptor activation and glutamate with decreases in intracellular calcium (38). The significance of these negative calcium responses is unclear, but an *in vivo* reduction in calcium signaling in striatal astrocytes increases self-grooming and reduces GABAergic tonic inhibition (39).

The nature of the decrease in cytoplasmic calcium is not well known. In these astrocytes, the increase in calcium triggered by glutamate may not be fast or robust but still sufficient to stimulate the activity of the ER Ca-ATPases, thereby decreasing cytoplasmic calcium. The close localization of SERCA, Na/Ca-exchangers, and IP3 receptors found in astrocytes could facilitate this process (40). Interestingly, several astrocytes that initially responded positively exhibited a decrease in calcium, followed by a rapid increase after glutamate exposure (Figure 2).

Analysis of calcium peaks revealed that positive and negative peaks of calcium had different behavior and dynamics. First, we found that positive and negative peaks correlated well with the AUCs, indicating that the size of peaks (both positive and negative) correlates closely with total calcium increase or decrease after glutamate administration. Interestingly, a few cells presented positive peaks with negative AUCs and vice versa. These cells exhibited small calcium peaks (Figure 4C and D), indicating a borderline balance between calcium entry, release, and extrusion mechanisms in these cells.

Negative peaks were smaller than positive peaks in the cells without TTX, but were not different in cells where glutamate was applied in the presence of TTX. Additionally, negative peaks had, on average, longer latencies and shorter durations (FWHM) than positive peaks. Notably, latencies showed no correlation with positive peak amplitudes. These differences highlight the distinct nature of the two types of responses, likely reflecting variations in the balance between calcium release and entry, as well as the processes involved in calcium clearance.

We concluded that NTS astrocytes exhibit heterogeneous responses to glutamate, with not all astrocytes showing a net increase in cytoplasmic calcium in response to external glutamate. A substantial fraction instead exhibited a decrease in intracellular calcium. These differing responses likely reflect a dynamic balance between calcium increases, mediated by release from internal stores and entry from the extracellular medium, and calcium clearance through endoplasmic reticulum-ATPases and mitochondria. Our findings reveal a more complex pattern of astrocytic responses to glutamate than previously assumed. Further investigations will be needed to elucidate the mechanisms underlying the intricate calcium dynamics in NTS astrocytes.

## Supplementary Materials

Supplementary Material Click to view [pdf].

## Data Availability

The original datasets generated during the current study are available from the corresponding author on reasonable request.
