# Supplementary material for: Complex dynamics of glutamate-induced calcium responses in astrocytes from the nucleus of the solitary tract of mice
Source: Braz J Med Biol Res. 2026 Jan 30;59:e15017. doi: 10.1590/1414-431X2025e15017 (PMC12858143; doi:10.1590/1414-431X2025e15017)

Figure S1. Electrophysiological recording of a SR101-labeled astrocyte (with permission from Drs. M.S. da Luz, D. Accorsi-Mendonça, and B.H. Machado).

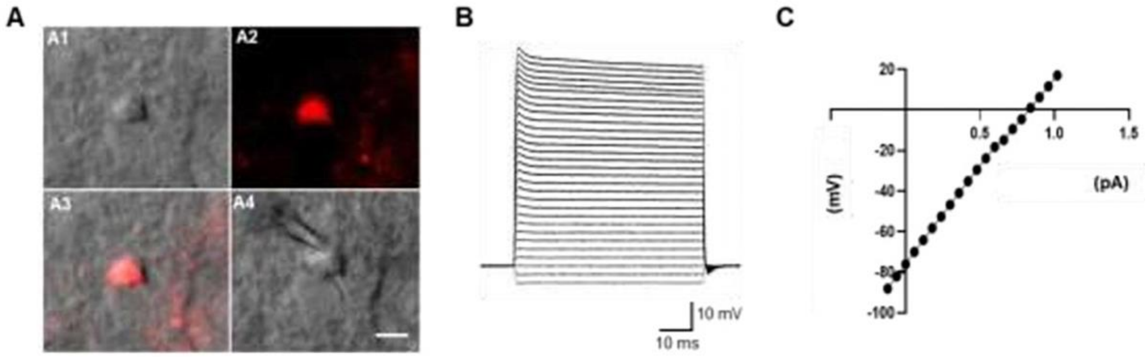

Supplement: Supplementary Material [file 1414-431X-bjmbr-59-e15017-suppl.pdf]
